# Reconstructing the COVID-19 incidence in India using airport screening data in Japan

**DOI:** 10.1186/s12879-023-08882-w

**Published:** 2024-01-02

**Authors:** Shiqi Liu, Asami Anzai, Hiroshi Nishiura

**Affiliations:** Kyoto University School of Public Health, Yoshidakonoe cho, Sakyo ku, Kyoto City, 6068501 Japan

**Keywords:** Border entry screening, severe acute respiratory syndrome coronavirus 2, Polymerase chain reaction, Statistical estimation, Epidemiology

## Abstract

**Background:**

A major epidemic of COVID-19 caused by the Delta variant (B.1.617.2) occurred in India from March to July 2021, resulting in 19 million documented cases. Given the limited healthcare and testing capacities, the actual number of infections is likely to have been greater than reported, and several modelling studies and excess mortality research indicate that this epidemic involved substantial morbidity and mortality.

**Methods:**

To estimate the incidence during this epidemic, we used border entry screening data in Japan to estimate the daily incidence and cumulative incidence of COVID-19 infection in India. Analysing the results of mandatory testing among non-Japanese passengers entering Japan from India, we calculated the prevalence and then backcalculated the incidence in India from February 28 to July 3, 2021.

**Results:**

The estimated number of infections ranged from 448 to 576 million people, indicating that 31.8% (95% confidence interval (CI): 26.1, 37.7) – 40.9% (95% CI: 33.5, 48.4) of the population in India had experienced COVID-19 infection from February 28 to July 3, 2021. In addition to obtaining cumulative incidence that was consistent with published estimates, we showed that the actual incidence of COVID-19 infection during the 2021 epidemic in India was approximately 30 times greater than that based on documented cases, giving a crude infection fatality risk of 0.47%. Adjusting for test-negative certificate before departure, the quality control of which was partly questionable, the cumulative incidence can potentially be up to 2.3–2.6 times greater than abovementioned estimates.

**Conclusions:**

Our estimate of approximately 32–41% cumulative infection risk from February 28 to July 3, 2021 is roughly consistent with other published estimates, and they can potentially be greater, given an exit screening before departure. The present study results suggest the potential utility of border entry screening data to backcalculate the incidence in countries with limited surveillance capacity owing to a major surge in infections.

**Supplementary Information:**

The online version contains supplementary material available at 10.1186/s12879-023-08882-w.

## Background

The severe acute respiratory syndrome coronavirus 2 (SARS-CoV-2) Delta variant (B.1.617.2) was first identified in India in late 2020 [1]. Soon after its emergence, India started to experience a rapid surge in cases of coronavirus disease 2019 (COVID-19) from early March to July, 2021, garnering global attention [2]. Genomic investigations indicated that the Delta variant nearly completely replaced previously circulating variants, including B.1.1.7 (Alpha), B.1.617.1 (Kappa), and others [3–5]. Even globally, the Delta variant had become dominant by mid-2021 [6]. Published laboratory studies indicated that the Delta variant possesses enhanced immune evasion capability and involves higher viral load than other variants [7–9]. Epidemiologically, these features are believed to have resulted in elevated transmissibility in comparison with wild-type SARS-CoV-2 and enhanced disease severity resulting in increased hospitalizations, especially among unvaccinated patients [10, 11]. The increased rate of hospital admissions led to serious shortages of care facilities (i.e., number of beds) as well as life-saving equipment and supplies, overwhelming the healthcare system in affected countries [10].

According to globally shared COVID-19 data from the Repository of the Center for Systems Science and Engineering at Johns Hopkins University [12], the number of newly documented cases (i.e., the reported number of cases, which may include cases that were not confirmed by RT-PCR or rapid diagnostic testing) per day on February 14, 2021, was on the order of 12,000 persons in India, declining from the highest recorded daily number of cases, 98,000 on September 16, 2020. Therefore, the government of India began to gradually relax non-pharmaceutical interventions and launched “Unlock 6.0” on October 27, 2020, thereby permitting resumption of outdoor activities [13, 14]. Published epidemiological studies have suggested that the lifting of restrictions on mass social gatherings, such as the Kumbh Mela festival in April 2021, may have caused a number of super-spreading events, exacerbating the second epidemic wave in India [15–17]. Although the national COVID-19 vaccination campaign in India began on January 16, 2021 [18], only 4.4% of the population had received the primary vaccination series (two doses) by July 3, 2021 [19]. From February 28 to July 3, 2021, India experienced a major epidemic and reported a total of 19,448,702 documented cases, which is twice the cumulative number of documented cases prior to that period. Moreover, a total of 244,954 deaths were documented, approximately 1.6 times more than the cumulative number of deaths up to that point, with an estimated daily case fatality risk ranging from 0.39 to 7.99%. The epidemic wave caused by the Delta variant led to a severe breakdown of the healthcare system in India, resulting in limited access to testing, shortages of hospital beds and ventilators, and overloading of morgues [20]. The fourth nationwide serosurvey revealed that approximately 67.6% of people aged ≥6 years in India had IgG antibodies against SARS-CoV-2 S1-RBD (subunits S1 of the Spike protein receptor binding domain) and/or nucleocapsid protein, which means that a large proportion of the population had developed immunity either owing to natural infection or vaccination by July, 2021 [21, 22]. However, only 4.4% of the population had received two doses of vaccine as of July 3, 2021, implying that the actual number of infections in India was far greater than reported.

Several epidemiological, demographic, and mathematical modelling studies have estimated the cumulative incidence of infections and mortality during the above-mentioned epidemic in 2021, characterizing the epidemiological and demographic features in India [3, 23–25]. For instance, an epidemiological study [23] estimated that 32.3% of the population in India had been infected with SARS-CoV-2 between late March and June 2021. That study used published (and documented) data from Johns Hopkins University, Google Community Mobility Reports, Our World in Data, and an epidemic model fitted to the temporal distribution of cases. Another study [24] applied a statistical approach to estimate the infection detection ratio, infection hospitalization ratio, and infection fatality risk (IFR) and analysed epidemiological datasets from Johns Hopkins University and additional data from local governments using a Bayesian cascading regression framework. The results of that study indicated that the cumulative incidence of infection was 64.3% from the start of the pandemic to November 4, 2021. A seroepidemiological study [3] found that by early July 2021, seropositivity had increased to 87.0% among unvaccinated individuals in Delhi, India. Another study [25] involved a national survey analysing all-cause mortality and comparing the rates of all-cause mortality between 2021 and 2020. From June 2020 to July 2021, 29% of total deaths in India—equivalent to 3.2 million people—were considered to have been caused by COVID-19, with 2.7 million deaths occurring during the COVID-19 surge from April to July 2021.

In the present study, we investigated the incidence of COVID-19 infection in India using border entry screening data in Japan, to estimate the daily incidence as well as cumulative incidence of infection in India during the Delta variant wave. Analysing the results of mandatory testing among non-Japanese passengers arriving from India entering Japan, we aimed to reconstruct the COVID-19 incidence in India during that period.

## Methods

### Entry screening in Japanese airports

Border control measures in Japan involved travel restrictions and entry/exit screening. The travel restrictions were realized by restricting visa, including visa suspension and regulation of visa types to permit an entry. From December 28, 2020, individuals holding new-entry visas from all countries were prohibited from entry. Although the restriction was only briefly eased for 23 days from November 8, 2021, the restriction restarted with the emergence of Omicron variant (B.1.1.529) by March 1, 2022. Only individuals holding re-entry visas and others (i.e., those holding family visas, diplomatic visas, or permanent resident visas) were allowed to enter Japan from September 1, 2020. Our study period, from February 28 to July 3, 2021, corresponded to the time period when individuals holding re-entry visas, family visas, and other certain visas were permitted to enter Japan. Even among re-entry visa holders, passengers from India were temporarily refused to enter due to growing epidemic of Delta variant (B.1.617.2) in India from May 14 to September 20, 2021.

Entry and exit screening were also strictly carried out. Exit screening measure mandated all passengers to Japan to present a negative test certificate of RT-PCR testing that was conducted within 72 hours before departure from January 13, 2021. During the epidemic wave of interest in 2021, entry screening was mandatorily conducted at all airports in Japan, with post-arrival testing of all incoming passengers carried out using real-time reverse transcription-polymerase chain reaction (RT-PCR). To conduct a large number of tests and post-hoc interviews among individuals with positive results as well as carry out quarantine procedures, airports that were open to international flight were restricted to Tokyo Narita, Tokyo Haneda, and Kansai International Airport, as officially planned by the Japanese government and implemented from April 3, 2020. Passengers were tested immediately upon arrival, and the testing result was available on-site in a matter of 2 hours. Any individuals who tested positive were either guided to begin isolation at designated hospitals or asked to remain at hotel facilities until recovery [26]. The results of entry screening were summarized according to passengers’ country of origin and nationality [27].

Due to different entry screening process between Japanese nationals and others, the present study used border entry screening data among non-Japanese people arriving from India for the period February 28 to July 3, 2021. The governmental data comprised weekly records for the number of RT-PCR tests conducted and the number of confirmed SARS-CoV-2-positive cases (see online Supplementary Table S1), enabling calculation of the positivity rate upon arrival. As indicated by the absence of positive cases among passengers arriving from zero-COVID countries [28], it is assumed that the infection event of positive passengers arriving from India mostly took place locally, i.e. in India. In the following analysis, we assumed that all travellers were randomly selected from among the general population of India.

### Additional datasets for statistical estimation

#### RT-PCR sensitivity and survival curve of test positivity

**Fig. 1:**
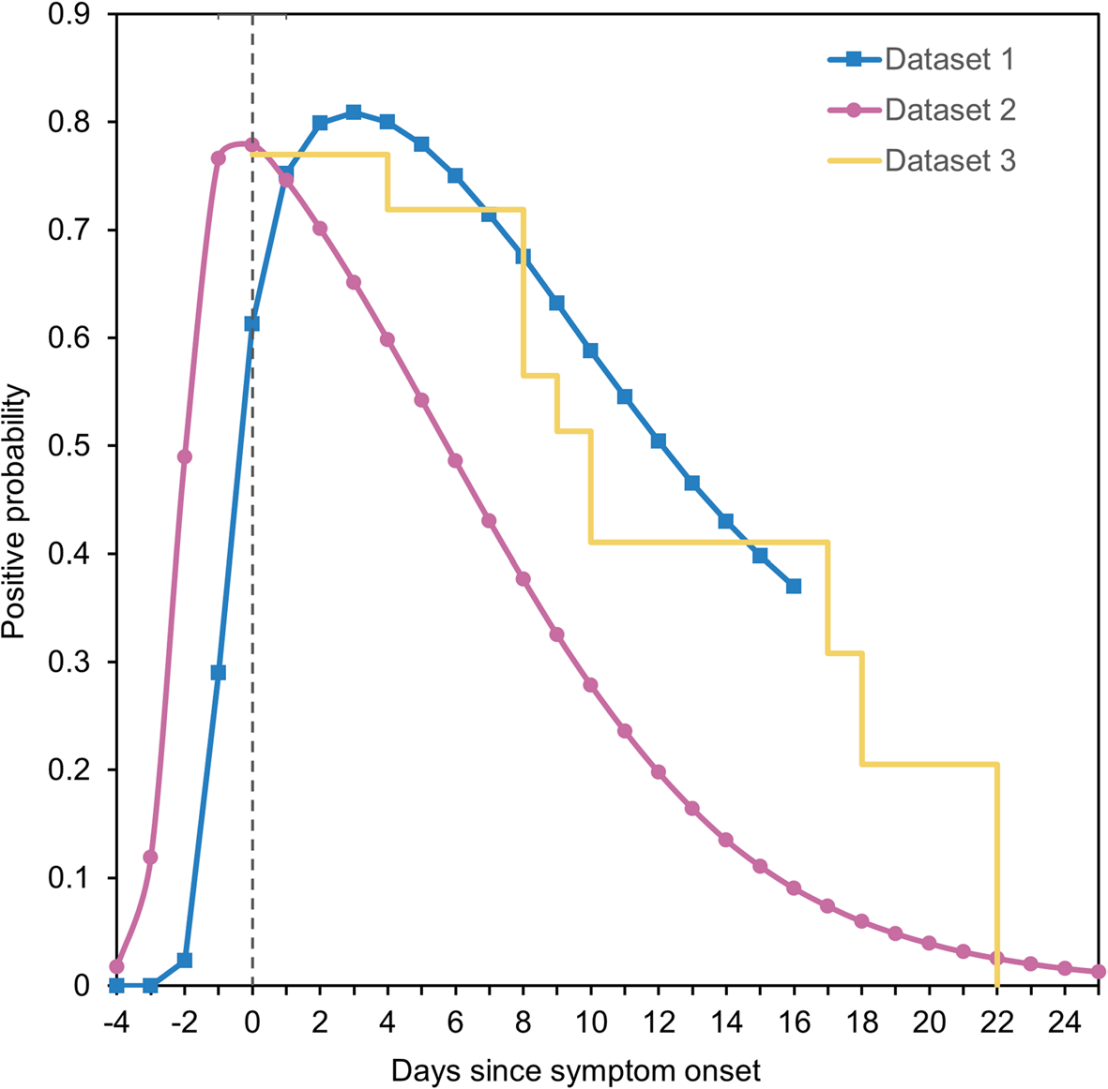
Empirical datasets used to convert from prevalence to incidence. Dataset 1 (blue line) exhibits the sensitivity of RT-PCR testing as a function of the time elapsed from the onset of symptoms [29]. The vertical dashed line denotes day 0 of symptom onset with the previous 5-day incubation period remaining unchanged from the original study. Dataset 2 (purple line) represents the probability of a positive RT-PCR test result, as estimated in a modelling study [30]. Dataset 3 is an assumed 77% sensitivity multiplied by a survival curve of RT-PCR positivity. The yellow line illustrates 77% sensitivity of RT-PCR positivity based on observation data from unvaccinated prisoners in the United States [31] with COVID-19 infection caused by the Delta variant

RT-PCR testing has been used as the gold standard in diagnosing SARS-CoV-2 infection. Nonetheless, the sensitivity of RT-PCR tests (i.e., true positive rate) can vary throughout the course of infection. During the early stages of infection when viral load is low, there is a high risk of false-negative results. In the middle stages of an infection when viral load is high, test sensitivity can reach the maximum. In the present study, we used data from Kucirka et al. [29] to address this issue; the data in this study was subject to the false negativity in RT-PCR results and time variation in the probability of detection We determined the probability of this combination of results as a function of days since exposure. To estimate the probability of changing sensitivity, we used the combined probability of false-negative results and the probability of detection. In the present study, we used published estimates on the sensitivity of RT-PCR as a function of time since exposure; most past studies tested samples at the time of symptom onset, which was assumed to have started on day 5 after exposure, as documented by Kucirka et al. [29]. As shown in Fig. 1, the sensitivity was 61.3% on the date of symptom onset (day 5 after exposure), and the sensitivity peaked at 80.9% on day 3 after onset (Fig. 1). The original data of Kucirka et al. were truncated at day 21 after exposure; accordingly, we truncated the distribution on that day (and dealt with the values on day 22 and later as zero).

As an alternative, we used a dataset from Hellewell et al. [30] to determine the probability of a positive RT-PCR result over the course of infection. Hellewell et al. applied a Bayesian model to estimate the probability among a cohort of 200 healthcare workers; the estimated peak probability was 77% at 4 days after infection, after which it began to decline.

Furthermore, we scaled the RT-PCR sensitivity by the probability of detection over time based on published estimates [30]. In the survival study of viremic period, a cohort of infected participants was followed up with repeated RT-PCR testing over the time after illness onset. To identify datasets of RT-PCR positivity for inclusion in the analysis, we used published data that satisfied the following conditions: (i) survival curve observed among individuals with mild COVID-19 infection or non-hospitalized individuals (i.e., not biased toward severe cases only), (ii) testing among individuals preferentially infected with the Delta variant, and (iii) individuals who remained unvaccinated. Accordingly, a study from the United States used RT-PCR data of unvaccinated prisoners with COVID-19 infection caused by the Delta variant [31]. That study provided a survival curve that started from the date of symptom onset. Analysis of the dataset revealed that the median duration of RT-PCR positivity was 17 days (Fig. 1).

#### Statistical estimation of incidence

Border entry screening data can be used to yield point prevalence estimates on a weekly basis. We aimed to reconstruct the COVID-19 incidence in India using these data. For this reason, we used the above-mentioned datasets to deconvolute the incidence, with the following equation:

1 $$p(t)=\sum_{k=0}^ti\left(t-k\right)f(k)\varGamma (k),\kern0.5em$$

where *p*(*t*) represents the prevalence of infection on day *t*; we assumed that RT-PCR positivity calculated using entry screening data mirrors this function. On the right-hand side, *i*(*t* − *k*) is the daily incidence that we wished to estimate. The RT-PCR sensitivity *f*(*k*) and survival curve of RT-PCR positivity *Γ*(*k*) were multiplied by the incidence; finally, convolution acts as a single-equation model to convert prevalence into incidence by deconvolving the equation.

As mentioned above, the empirical data of entry screening was summarized according to the week of observation; thus, it was not feasible to precisely estimate the daily incidence. For this reason, we decided to take advantage of the discrete data and assumed that the prevalence and incidence were in quasi-equilibrium within each single reporting interval (i.e., prevalence and incidence took constant values every 2 weeks), denoted by p* and i*. This assumption allowed us to have

$${i}^{\ast }(t)\cong {i}^{\ast}\left(t-1\right)\cong {i}^{\ast}\left(t-2\right)\dots \cong {i}^{\ast}\left(t-k\right)$$

and

$${p}^{\ast }(t)={i}^{\ast }(t)\sum_{k=0}^tf(k)\varGamma (k),$$

yielding an estimator

2 $${i}^{\ast }(t)=\frac{p^{\ast }(t)}{\sum_{k=0}^tf(k)\varGamma (k)}.$$

In this approximation, we can consider that the prevalence p* divided by the sum of *f*(*k*)*Γ*(*k*), which was assumed to be equal to the daily incidence in the corresponding weekly interval. In fact, the product of *f*(*k*)*Γ*(*k*) is assumed to be represented by one of the three empirical datasets mentioned above.

The maximum likelihood method was used to compute the weekly prevalence using a binomial distribution, which also informs the uncertainty bound of the daily incidence (assuming constant daily incidence for every 2 weeks) using eq. (2). Subsequently, on the basis of the obtained daily incidence results, cumulative incidence of infection was computed, and confidence interval of the incidence was calculated using the parametric bootstrap method. While obtaining the estimate, it should be noted that the present study did not impose any specific assumption over re-infection; while a part of published studies implied the presence of re-infection [3, 23, 32], a large-scale analysis in South African indicated the absence of re-infection [33]. Comparing the estimate against reported values, ascertainment ratios were computed over the course of time. The ascertainment ratio was defined as the ratio of the estimated number of COVID-19 infections to the documented number of cases. The 95% confidence intervals (CIs) of daily incidence were computed using the profile likelihood. When illustrating the prevalence, its 95% CIs were calculated using the Wilson score method.

#### Adjustment for exit screening

Considering that only test-negatives can board on flight, the positivity rate from airport screening may be an underestimate compared with the actual prevalence in India. Exit screening enforced all travelers to submit test-negative certificate obtained within 72 hours before departure, but despite the strict rule, it is widely recognized in India that there were substantial number of passengers presenting pre-departure negative test certificates that did not meet the testing quality standards, and moreover, falsification of pre-departure certificates existed [34, 35]. Although we cannot strictly adjust those validity issues without corresponding dataset, here we considered an alternative (adjusted) prevalence accounting for the proportion of testing that cannot be trusted. Let *P* be the estimated cumulative incidence based on entry screening data in Japan and *ϵ* (=0.7) be the test sensitivity. Suppose that the fraction *η* is questionable certificate (e.g. illegitimate testing or even fake certificate), the probability that exit screening gave a false negative result is (1-ϵη). As we discussed in Eq. (2), *f*(*z*)*Γ*(*z*) corresponds to loss of positivity during travel of the time length *z*, and *f*(72 *h*)*Γ*(72 *h*) = 0.70 if we used the healthcare worker data from the UK [30] and *f*(72 *h*)*Γ*(72 *h*) = 0.77 if we used the American prisoner data [31]. Let *B* be the adjusted cumulative incidence in India, and let *q* represent the propensity that travelers with test-negative certificates are less likely infected than the general population in India, we have an adjusting equation:

3 $$B=\frac{P}{q\left(1-\epsilon \eta \right)f(z)\varGamma (z)}.$$

As we cannot derive plausible value of *η*, the estimate of B was computed for the range of *η* from 0 to 1. Similarly, the actual value of *q* has never been directly measured, and for the exposition of biased risk of infection among travelers, we used q = 0.8 for the sake of illustration only.

## Results

**Fig. 2:**
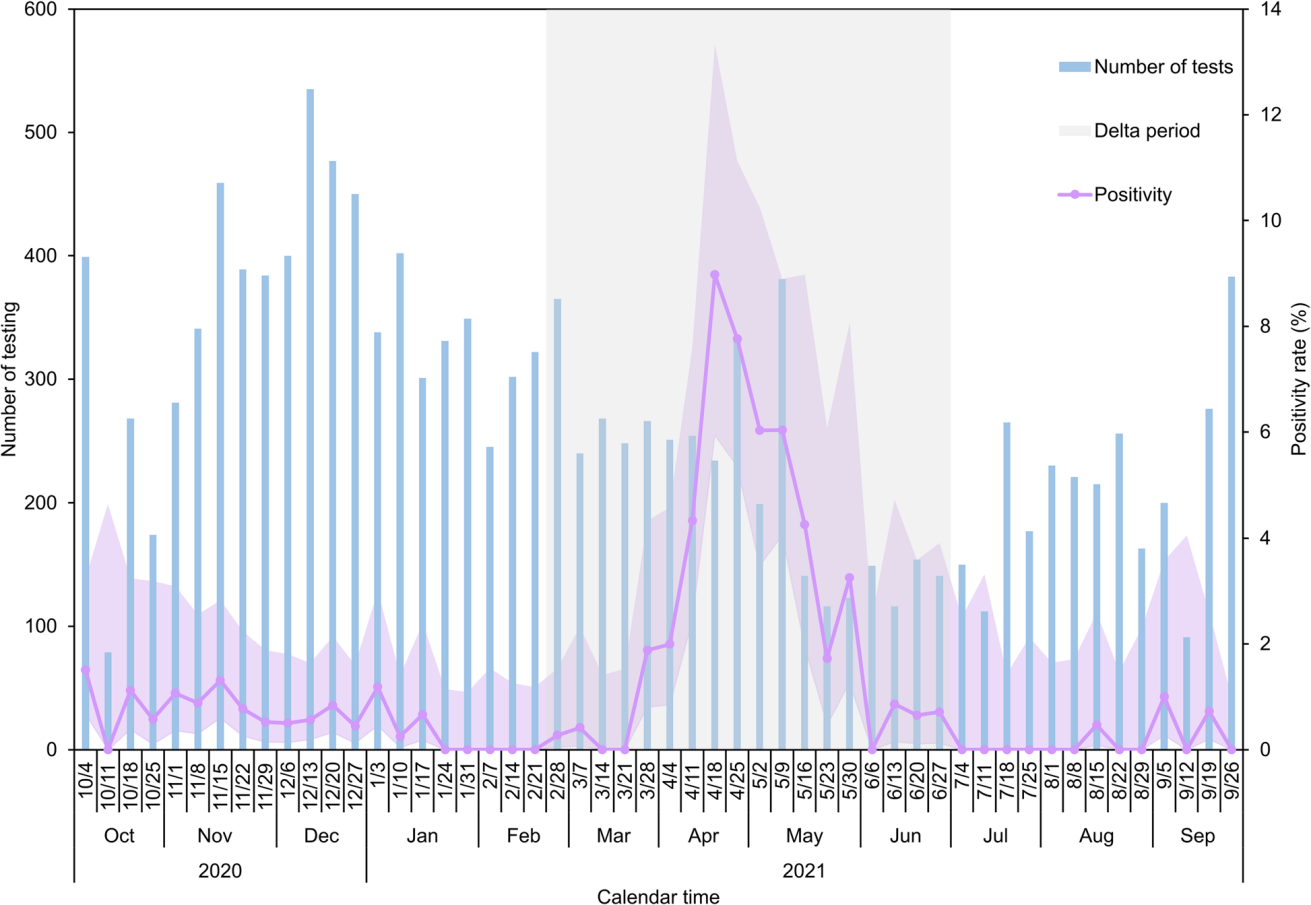
Entry screening data in Japan among Indian passengers from India from October 4, 2020 to October 2, 2021. The blue bars represent the weekly number of RT-PCR tests conducted for all incoming non-Japanese passengers from India entering Japan. The pink line with markers represents the corresponding weekly proportion with positive results. The pink-shaded area represents the upper and lower 95% confidence interval of the proportion with positive results. The grey-shaded area indicates the study period (February 28 to July 3, 2021) during the epidemic caused by the Delta variant

From February 28 to July 3, 2021, a total of 3981 RT-PCR tests were carried out at international airports in Japan for non-Japanese passengers arriving from India, resulting in 120 positive results for SARS-CoV-2, and yielding an overall positivity rate of 3.0% (95% CI: 2.5, 3.6). The airport entry screening data revealed a significant surge in the rate of positivity from the week beginning on March 28, 2021, which reached its peak at 9.0% (95% CI: 5.9, 13.3) in the week beginning on April 18, 2021, followed by a gradual decline (Fig. 2). On May 14, 2021, the Japanese government began to prohibit foreign passengers holding re-entry visas from India to enter the country. Even prior to that decision, new-entry passengers had not been allowed to enter Japan.

**Fig. 3:**
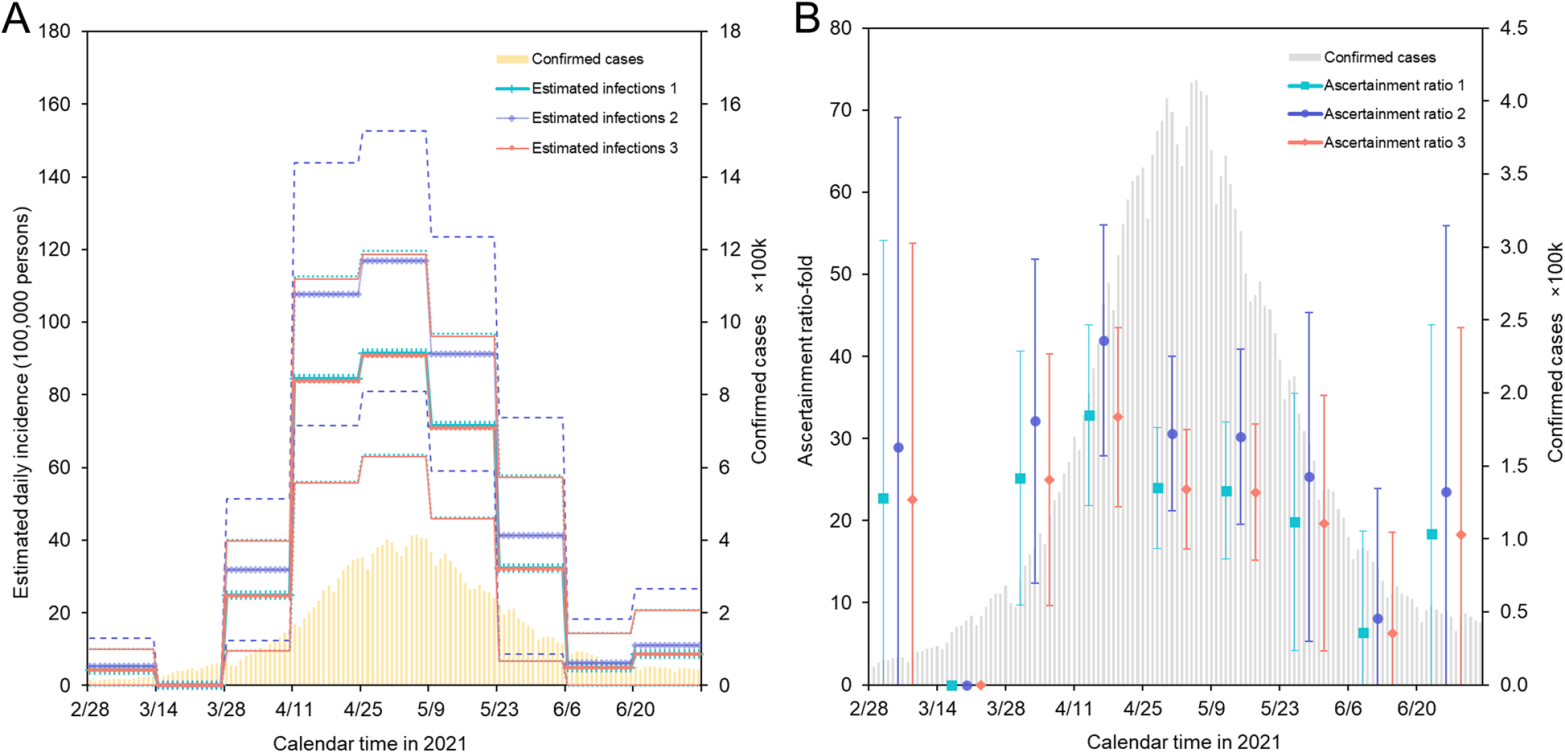
Reconstructed epidemic curve and ascertainment ratio in India, 2021. **A** Estimated daily incidence of infection overlaid with officially reported number of documented cases by the government of India over the period from February 28 to July 3, 2021. Two vertical axes are calculated with the unit of 100,000 persons for the entire country of India. The green line with sticks represents estimated infections using dataset 1 of Kucirka et al. [29]. The dotted lines show the 95% confidence intervals (CI); however, this is mostly overlapped with estimates using dataset 3. The purple with diamond markers represents estimated infections using dataset 2 of Hellewell et al. [30] accompanied by 95% CI represented by dashed lines. Estimated infections using dataset 3 are derived using data of unvaccinated prisoners during an epidemic wave caused by the Delta variant in the United States, represented by the orange line with solid lines for the 95% CI [31]. Estimation 1 and estimation 3 were right-overlapped with each other. **B** Ascertainment ratio over time, calculated as the biweekly number of estimated infections over the biweekly reported number of documented cases; each observation period has three different estimates using three different datasets. The left-hand vertical axis represents the ascertainment ratio, and the right-hand vertical axis represents the number of documented cases. Ascertainment ratios 1, 2, and 3 correspond to estimates using datasets 1, 2, and 3, respectively

Figure 3A shows the estimated daily incidence of infection using three datasets of *f*(*k*)*Γ*(*k*), overlaid with officially documented cases in India. Two estimated curves based on Kucirka et al. and American prisoner data showed similar qualitative patterns, with only small variations, except for one curve using data of Hellewell et al. that showed a different trend. Those data showed that the estimated curves peaked during the week of April 25, with a daily incidence ranging from 0.6% (95% CI: 0.4, 0.8%) to 0.8% (95% CI: 0.6, 1.1%), which corresponded to 9.1–11.7 million infections per day (using a population estimated of 2021 from the United Nations [36]). In contrast, the highest number of documented cases, involving the incubation period and delays in diagnosis and reporting, was reported by the Indian government on May 6, with the peak seen in the week beginning on May 2, 2021.

Our estimate of the number of SARS-CoV-2-infected individuals was approximately 24 (95% CI: 17, 31) to 31 (95% CI: 21, 40) times greater than the reported number of documented cases recorded in the peak weeks (Fig. 3B). From February 28 to July 3, 2021, India reported 19.4 million documented cases, corresponding to 1.4% of the population in India during 2021. However, according to our estimation, the number of infections ranged from 448 to 576 million people, indicating that 31.8% (95% CI: 26.1, 37.7%) to 40.9% (95% CI: 33.5, 48.4%) of the population had been infected from February 28 to July 3, 2021. These figures indicate that the actual number of infected individuals was 23 to 30 times greater than the documented number of cases. The upper bound of our estimate suggested that approximately 40.9% of the population experienced infection. The ascertainment ratio, as of February 28, showed an estimated 23 (95% CI: 0, 54) to 29 (95% CI: 0, 69)-fold, and in the following weeks, the ascertainment ratio ranged from 25- to 42-fold (except for the week starting on March 14 where the ratio was zero). By April 11, 2021, the ascertainment ratio reached its peak value of a 33 (95% CI: 22, 44) to 42 (95% CI: 28, 56) -fold, and this occurred just before the peak of estimated incidence. After the peak, the ascertainment ratio declined and ranged from 6 (95% CI: 0, 19) to 8 (95% CI: 0, 24) -fold during the week of June 6, 2021.

**Fig. 4:**
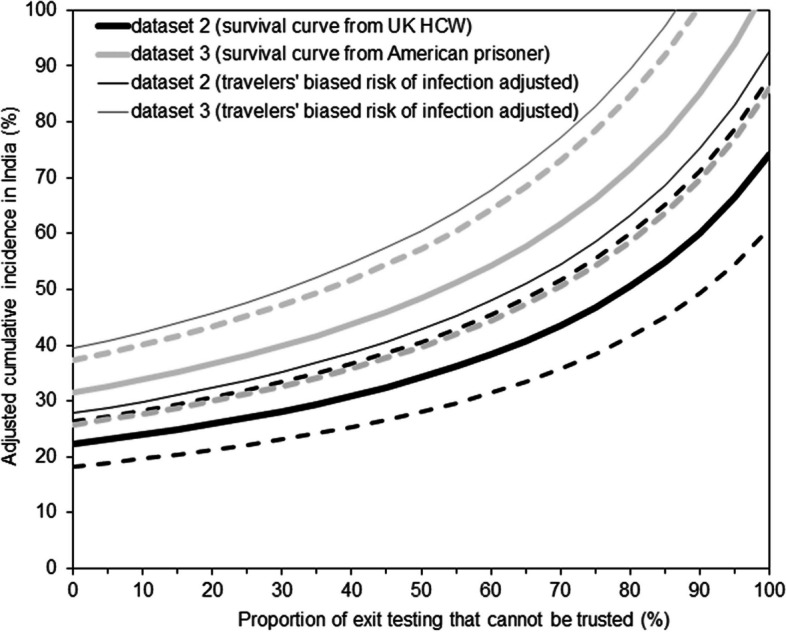
Bias adjusted cumulative incidence in India, 2021. The adjusted cumulative incidence is shown as a function of the proportion of exit screening in India that cannot be trusted. Discarding the biased risk of infection among travelers (i.e., *q* = 1), thick black lines show the estimate from dataset 2 among healthcare workers in the United Kingdom [30], while thick grey lines show estimates from dataset 3 derived from American prisoners [31]. Dashed lines represent the 95% confidence intervals that were obtained during the original estimation with *q* = 1. Thin black and grey continuous lines show estimates from datasets 2 and 3, respectively, when we assume that the risk of infection among travelers was 20% smaller than the general population in India (i.e., *q* = 0.8)

Correcting questionable or untrustworthy test-negative certificates with *q* = 1, the cumulative incidence is elevated for up to 2.3–2.6 times the estimates that we have described above (Fig. 4). For instance, if 50% of negative certificate was questionable, the adjusted cumulative incidence would be 34.2 and 48.5% for datasets 2 and 3, respectively, in contrast to 31.8 and 40.9% as the original underestimate. Similarly, if 75% questionable, the cumulative incidence may be as high as 46.9 and 66.3%, respectively, for datasets 2 and 3. That is, rather than ascertainment ratio of 23–30 times reported values, involving questionable test-negative certificates leads to an adjusted ascertainment ratio of 54–77 times reported values. When *q* = 0.8, all of those mentioned above would be scaled up by the factor of 1.25.

## Discussion

In the present study, we estimated the incidence of COVID-19 during the Delta variant epidemic in India during 2021, using airport entry screening data from Japan. The analysis was conducted over a period of 18 weeks, from February 28 to July 3, 2021. The entry screening data suggested that a substantial proportion of the Indian population was infected during the corresponding period, with an estimated cumulative risk of infection of approximately 40.9%. Notably, the highest daily incidence was observed from April 25, with an estimated 11.7 million infections per day and a daily incidence rate of 0.8%. Furthermore, the overall ascertainment ratio reached a 30-fold over the observed documented cases. Accounting for mandatory test-negative certificate as an exit screening, we additionally carried out possible adjustment of cumulative incidence. Although a part of test-negative certificates were questionable, the cumulative incidence can potentially be up to 2.3–2.6 times greater than abovementioned estimates. If we further account for the biased risk of travelers, the prevalence of the general population would be even greater. We also found that the estimated epidemic peak occurred from late April, approximately 1 week earlier than the peak in the number of documented cases in early May, which is consistent with the sum of the mean incubation period and mean time delay from illness onset to reporting [37, 38].

As a take home message, the present study showed that border entry screening-based prevalence can be used to help reconstruct the incidence in the origin country. Our estimate of approximately 40.9% cumulative infection risk from February 28 to July 3, 2021 is roughly consistent with the 32.3% obtained in a modelling method [23] and the difference could be explained by different study period (e.g. the modelling study [23] explored from late March to June, while our study covered up to July 3, 2021). Our estimate was smaller than the estimated 64.3% [24] from the start of the pandemic to November 14, 2021; that study reported cumulative incidence for the entire period up to November 2021. Considering that the cumulative percentage of documented cases before March 2021 was 0.79% (approximately 11 million) of the population in India and our overall ascertainment ratio ranged from 23 to 30 times, a 40% estimated cumulative risk of infection can be considered reasonable. In addition, our adjustment indicated that the presence of exit-screening led us to potentially underestimate the actual cumulative incidence by up to 2.3–2.6 times. During the study period, the case fatality ratio among documented COVID-19 cases was calculated at 1.26%, but both cases and deaths were considerably under-ascertained. To address this issue, excess mortality studies [39, 40] have been conducted. Especially in India, another study [25] using a national survey and health facility data estimated that 2.7 million deaths occurred in India from April to July, 2021. The 2.7 million deaths and our estimate of 40.9% infections yields an IFR of 0.47%. This is not far from the IFR of 0.3% in the above-mentioned study [24] as of November 4, 2021.

The present study results suggest the potential utility of border entry screening data to backcalculate the incidence in countries with limited surveillance capacity owing to a major surge in infections. However, an inherent assumption that had to be imposed was a random sample from the origin country, which may not be true for three reasons. First, infection frequently involves heterogeneity. For instance, if economically disadvantaged people are more vulnerable to infection than other groups, and if this high-risk strata of the population cannot afford to travel internationally, biased sampling can occur and our results might have been underestimated. In fact, the epidemic is known to have been initially geographically heterogeneous and very intense transmission rate was indicated in Maharashtra [41, 42]. Second, human travel behaviour is somewhat related to infection events. If exposure occurs shortly before departure or if suspicious symptoms occur prior to the departure time, an individual may cancel their travel plans. Third, travelers were less likely to be experiencing COVID-19 symptoms, so the travelers tested by the border screening are likely over-represented by asymptomatically infected or uninfected individuals, who have a lower positivity than the general public, leading to underestimation of incidence as well [43]. Again, we may have underestimated the incidence if only healthy individuals were sampled as international travellers. Nevertheless, it could also be the case that, given an uncontrollable surge in COVID-19 cases caused by the Delta variant, people at risk may have travelled from India to other countries with a lower risk. Such evacuation behaviour introduces an opposite bias to elevate the risk of infection among travellers. At minimum, we have seen that border entry screening data among people from the United Kingdom were consistent with the magnitude and temporal patterns according to results of a prevalence survey conducted by the Office of National Statistics COVID-19 Infection (Nishiura, personal communication); thus, we believe that the overall magnitude and temporal patterns of COVID-19 infection in India were well captured.

Despite the methodological uniqueness of using border entry screening data, six limitations should be discussed. First, during our study period, individuals with re-entry visas were allowed entry into Japan, and this group may not be representative of the general Indian population, potentially leading to an underestimate of the cumulative incidence. Second, mandatory testing policy was underway during the period of study. Prior to the study period on January 13, 2021, Japan had explicitly requested exit screening, mandating that all incoming passengers undertake RT-PCR testing within 72 hours before departure and submit a certificate of the negative result. People who tested positive or developed symptom were refused to board their flight to reduce the risk of infecting other airline passengers, and for this reason, only the people who tested negative were allowed to depart, imposing unavoidable selection bias in the data due to exit screening. Nevertheless, the validity of RT-PCR testing results was seriously questioned during the Delta variant epidemic [34, 35], and at least we addressed the abovementioned points via simulations (Fig. 4). Third, we did not explicitly account for the time delay required for international flights. That is, because international travel takes a longer time, it becomes more likely that travellers were in the incubation period of SARS-CoV-2 infection and developed illness later. This point was taken into account in our adjustment of cumulative incidence (Fig. 4). Fourth, we used the days from symptom onset to model RT-PCR test sensitivity assuming a constant incubation period of 5 days. The three datasets used showed different variation in sensitivity over the course of time since infection. Estimate using dataset 3 yielded the lowest results compared with two other datasets. Fifth, in the present study, we managed to estimate the incidence level for the entire population, although it was plausible that the obtained incidence was an underestimate. Building on such evidence, it would be ideal to reconstruct the epidemic dynamics across ages and geographic space. Combining the screening results with additional local epidemiological datasets is the subject of future research. Sixth, the number of passengers testing positive for COVID-19 was limited, and our simple conversion from prevalence to incidence did not explicitly address the incidence estimation during weeks with zero positive test results.

## Conclusion

In the present study, we used border entry screening data in Japan to backcalculate the COVID-19 incidence in India. Approximately, 40.9% of the population of India was estimated to have experienced SARS-CoV-2 infection during the Delta variant wave in 2021. We not only obtained cumulative incidence that is consistent with published estimates, but also showed that the actual incidence of infection was estimated to be 30 times greater than that based on documented cases, giving a crude IFR of 0.47%. Adjusting for test-negative certificate before departure, the quality control of which was partly questionable, the cumulative incidence can potentially be up to 2.3–2.6 times greater than abovementioned estimates.

### Supplementary Information

**Additional file 1.**

## Data Availability

Airport entry screening data among non-Japanese passengers arriving from India are publicly available on the website of the Ministry of Health, Labour and Welfare [27], and a translated version in English is provided as Supplementary Table S1.

## Abbreviations

COVID-19: novel coronavirus disease 2019
ARS-CoV-2: Severe Acute Respiratory Syndrome Coronavirus 2
RT-PCR: real-time reverse transcription-polymerase chain reaction
