# Supplementary material for: Reconstructing the COVID-19 incidence in India using airport screening data in Japan
Source: BMC Infect Dis. 2024 Jan 2;24:12. doi: 10.1186/s12879-023-08882-w (PMC10763058; doi:10.1186/s12879-023-08882-w)
Supplement: Supplementary file 1 — Additional file 1. [file 12879_2023_8882_MOESM1_ESM.docx]

**Supplementary Information**

The online version contains supplementary material available at

Additional file 1: Table S1. The airport entry screening data comprised weekly records for the number of RT-PCR tests conducted and the number of confirmed SARS-CoV-2-positive cases.

**Supplementary Table S1**

| Week | Time Period | Number of tests | Number of positive cases |
| --- | --- | --- | --- |
| 1 | 10/4-10/10, 2020 | 399 | 6 |
| 2 | 10/11-10/17, 2020 | 79 | 0 |
| 3 | 10/18-10/24, 2020 | 268 | 3 |
| 4 | 10/25-10/31, 2020 | 174 | 1 |
| 5 | 11/1-11/7, 2020 | 281 | 3 |
| 6 | 11/8-11/14, 2020 | 341 | 3 |
| 7 | 11/15-11/21, 2020 | 459 | 6 |
| 8 | 11/22-11/28, 2020 | 389 | 3 |
| 9 | 11/29-12/5, 2020 | 384 | 2 |
| 10 | 12/6-12/12, 2020 | 400 | 2 |
| 11 | 12/13-12/19, 2020 | 535 | 3 |
| 12 | 12/20-12/26, 2020 | 477 | 4 |
| 13 | 12/27-1/2, 2020 | 450 | 2 |
| 14 | 1/3-1/9, 2021 | 338 | 4 |
| 15 | 1/10-1/16, 2021 | 402 | 1 |
| 16 | 1/17-1/23, 2021 | 301 | 2 |
| 17 | 1/24-1/30, 2021 | 331 | 0 |
| 18 | 1/31-2/6, 2021 | 349 | 0 |
| 19 | 2/7-2/13, 2021 | 245 | 0 |
| 20 | 2/14-2/20, 2021 | 302 | 0 |
| 21 | 2/21-2/27, 2021 | 322 | 0 |
| 22 | 2/28-3/6, 2021 | 365 | 1 |
| 23 | 3/7-3/13, 2021 | 240 | 1 |
| 24 | 3/14-3/20, 2021 | 268 | 0 |
| 25 | 3/21-3/27, 2021 | 248 | 0 |
| 26 | 3/28-4/3, 2021 | 266 | 5 |
| 27 | 4/4-4/10, 2021 | 251 | 5 |
| 28 | 4/11-4/17, 2021 | 254 | 11 |
| 29 | 4/18-4/24, 2021 | 234 | 21 |
| 30 | 4/25-5/1, 2021 | 335 | 26 |
| 31 | 5/2-5/8, 2021 | 199 | 12 |
| 32 | 5/9-5/15, 2021 | 381 | 23 |
| 33 | 5/16-5/22, 2021 | 141 | 6 |
| 34 | 5/23-5/29, 2021 | 116 | 2 |
| 35 | 05/30-06/05, 2021 | 123 | 4 |
| 36 | 06/06-06/12, 2021 | 149 | 0 |
| 37 | 06/13-06/19, 2021 | 116 | 1 |
| 38 | 06/20-06/26, 2021 | 154 | 1 |
| 39 | 06/27-07/03, 2021 | 141 | 1 |
| 40 | 07/4-07/10, 2021 | 150 | 0 |
| 41 | 07/11-07/17, 2021 | 112 | 0 |
| 42 | 07/18-07/24, 2021 | 265 | 0 |
| 43 | 07/25-07/31, 2021 | 177 | 0 |
| 44 | 08/01-08/07, 2021 | 230 | 0 |
| 45 | 08/08-08/14, 2021 | 221 | 0 |
| 46 | 08/15-08/21, 2021 | 215 | 1 |
| 47 | 08/22-08/28, 2021 | 256 | 0 |
| 48 | 08/29-09/04, 2021 | 163 | 0 |
| 49 | 09/05-09/11, 2021 | 200 | 2 |
| 50 | 09/12-09/18, 2021 | 91 | 0 |
| 51 | 09/19-09/25, 2021 | 276 | 2 |
| 52 | 09/26-10/02, 2021 | 383 | 0 |
